# Sense of coherence as a resource in relation to health-related quality of life among mentally intact nursing home residents – a questionnaire study

**DOI:** 10.1186/1477-7525-6-85

**Published:** 2008-10-21

**Authors:** Jorunn Drageset, Harald A Nygaard, Geir Egil Eide, Margareth Bondevik, Monica W Nortvedt, Gerd Karin Natvig

**Affiliations:** 1 Faculty of Health and Social Sciences, Bergen University College, Haugeveien 28, N-5005 Bergen, Norway; 2 Department of Public Health and Primary Health Care, University of Bergen, Kalfarveien 31, N-5018 Bergen, Norway; 3 NKS Olaviken Hospital for Old Age Psychiatry, N-5306 Erdal and Section for Geriatric Medicine, Department of Public Health and Primary Health Care, University of Bergen, Norway; 4 Centre for Clinical Research, Haukeland University Hospital, N-5021 Bergen, Norway

## Abstract

**Background:**

Sense of coherence (SOC) is a strong determinant of positive health and successful coping. For older people living in the community or staying in a hospital, SOC has been shown to be associated with health-related quality of life (HRQOL). Studies focusing on this aspect among nursing home (NH) residents have been limited. This study investigated the relationship between SOC and HRQOL among older people living in NHs in Bergen, Norway.

**Methods:**

Based on the salutogenic theoretical framework, we used a descriptive correlation design using personal interviews. We collected data from 227 mentally intact NH residents for 14 months in 2004–2005. The residents' HRQOL and coping ability were measured using the SF-36 Health Survey and the Sense of Coherence Scale (SOC-13), respectively. We analyzed possible relationships between the SOC-13 variables and SF-36 subdimensions, controlling for age, sex, marital status, education and comorbidity, and investigated interactions between the SOC and demographic variables by using multiple regression.

**Results:**

SOC scores were significantly correlated with all SF-36 subscales: the strongest with mental health (*r *= 0.61) and the weakest with bodily pain (*r *= 0.28). These did not change substantially after adjusting for the associations with demographic variables and comorbidity. SOC-13 did not interact significantly with the other covariates.

**Conclusion:**

These findings suggest that more coping resources improve HRQOL. This may indicate the importance of strengthening the residents' SOC to improve the perceived HRQOL. Such knowledge may help the international community in developing nursing regimens to improve HRQOL for older people living in NHs.

## Background

Similar to other countries in Europe [1,2], nursing homes (NHs) in Norway are part of the public health care system and are intended for the long-term care of frail, older people. In other countries such as the United States, NHs may also be private institutions [1]. In Norway, and in other countries, a number of beds in NHs are allocated for respite and for rehabilitation [1,3]. In addition, most NHs offers regular units or a special care unit for people with dementia [1,3]. Long-term care facilities aim to provide care that enables residents to attain or maintain their maximal functional capacity [4] and health-related quality of life (HRQOL) [3,4]. NHs are intended for any person in need of long-term care that the home nursing services cannot deliver. However, about 80% of NH residents have dementia [6]. In addition to multiple diagnoses, many NH residents have experienced other stressful events such as loss of home and relational losses.

It is therefore important not only to study the residents' limitations but also to examine their resources and strengths in relation to coping with loss and to study why older people may manage well despite impaired physical capacity and adversity. Thus, this study explored the idea that focusing on resources and capacity is more important than focusing on disease and/or impairment in promoting healthy well-being among older people.

Antonovsky [7,8] examined health-promoting factors in his salutogenic model and developed the concept of sense of coherence (SOC) to explain why some people become ill when stressed while others remain healthy. SOC is defined as "global orientation that expresses the extent to which one has a pervasive, enduring though dynamic feeling of confidence" [[8], p. 19]. SOC generally expresses an individual view of the world and has three components: comprehensibility (the extent to which stimuli from one external and internal environment are structured, explicable and predictable) manageability (the extent to which resources are available to a person to meet the demands posed by these stimuli) and meaningfulness (the extent to which these demands are challenges worthy of investment and engagement). According to Antonovsky [8], people who have developed a strong SOC tend to perceive their situation as understandable, manageable and meaningful. Strong SOC suggests that an individual possesses resources (such as social support and ego identity) that enable the person to cope with various kinds of stressful life events. According to Antonovsky [8], people who have developed a strong SOC tend to perceive their situation as understandable, manageable and meaningful. He contends on a theoretical basis that SOC is relatively stabilized by the end of young adulthood and is thereafter affected only slightly positively or negatively by major life events. However, recent empirical findings suggest inconsistency regarding how SOC varies by age. Specifically, Nilsson et al. [9], Ekman et al. [10] and Nygren et al. [11] have shown that SOC tends to increase with age, whereas Borglin et al. [12] found that SOC decreases with age. Moreover, some researchers [9,13] have reported no significant differences in SOC between men and women, whereas others [14] reported that men had higher SOC than women.

Several studies [10,11,15-18] have shown positive associations between SOC and HRQOL among older people living in the community or staying in a hospital. Some [10,11,15] used the SF-36 Health Survey to measure HRQOL. A study among participants aged 85 years and older living at home [11] found no significant relationship between SOC and the SF-36 physical summary scale among men or women. However, SOC was significantly correlated with the SF-36 mental summary scale. High SOC was related to high HRQOL among older patients with angina (mean age 66 years) [15]. Although studies have reported positive relationships between SOC and HRQOL, to our knowledge no study has examined the relationship between SOC and HRQOL among NH residents. Many NH residents have low physical functioning [19,20]. It is therefore of interest in this population to investigate whether physical functioning and SOC are strongly related or whether the coping in this population is related to other aspects of HRQOL. Our study included subjects living in long-term care with multiple diagnoses, and the only similar study was among hospitalized patients needing acute hospital care (mean age 81 years, range 65–96 years) with only one defined diagnosis: chronic heart failure [10]. The results showed significant positive associations between SOC and all SF-36 subdimensions except for bodily pain and social functioning. We believe that this makes this study important.

Dementia care in NHs has attracted great interest during the past decade due to the great challenge this group of people represents. Mentally intact NH residents constitute a minority, and their needs have largely been given less priority. We have previously shown that mentally intact NH residents have markedly reduced HRQOL assessed using SF-36 compared with the general population of the same age and sex [21]. In the present study, we wanted to examine the relationship between SOC and HRQOL among mentally intact NH residents. Such knowledge may help the international community in developing nursing regimens to improve HRQOL for older people living in NHs.

Based on a review of the previous research on SOC and HRQOL and on Antonovsky's theory, the aims of the study were to assess the relationship between SOC and the SF-36 subdimensions in mental intact NH population and to investigate whether level of education, age, sex, marital status and comorbidity modify this relationship.

## Methods

### Design

This study used a cross-sectional, descriptive, correlational design.

### Sample

Long-term care residents from all 30 NHs in Bergen, Norway were potential participants. We collected data between 15 January 2004 and 31 May 2005. Our sampling frame included all residents who were ≥ 65 years, mentally intact and capable of carrying out a conversation and had been residing in the NHs for at least 6 months. We defined mentally intact as having a Clinical Dementia Rating (CDR) ≤ 0.5 [22], which was assessed by trained nurses who knew the residents well. In this context, we classified CDR as: mentally intact (CDR = 0); senescent forgetfulness (CDR = 0.5); and mild (CDR = 1), moderate (CDR = 2) or severe mental impairment (CDR = 3) [22]. A previous study showed excellent agreement between trained nurses' evaluation of mental capacity based on CDR and the diagnosis of dementia [23]. Of 2042 NH residents, 252 fulfilled the inclusion criteria, and a primary care nurse invited them to participate. Of these, 25 (10%) refused to participate. For those who agreed to participate (*n *= 227, 90%), we obtained the data through face-to-face interviews. The interview took place in the respondent's room or at another appropriate location in the nursing home. The principal investigator (JD) recorded the demographic information and performed the interviews: that is, reading the questions to the participants and circling the indicated answer. This was necessary, as many of the residents have problems holding a pen and have reduced vision. Each participant received a large-type version of the questionnaire so they could follow the questions. The principal investigator ensured that the questions were understood. Thus, the NH sample comprised 227 residents for data collection and analysis.

The Western Norway Committee for Medical Research Ethics approved the study protocol and consent procedures. All participants provided written informed consent. The Norwegian Social Science Data Services approved the study.

### Measures

#### Demographic and comorbidity variables

Sociodemographic data such as age, sex, marital status and educational level were collected. Comorbidity assessed using the Functional Comorbidity Index (FCI), a clinically based measure developed by Groll et al. [24]. This index includes 18 diagnoses scored "yes = 1" and "no = 0". A maximum score of 18 indicates the highest number of comorbid illnesses.

#### The Sense of Coherence Scale

The Sense of Coherence Scale (SOC-13) was used to estimate the resident's SOC. The scale has a 7-point Likert scale format with two anchoring responses, "never" and "very often". The items measured were perceived comprehensibility (5 items), manageability (4 items) and meaningfulness (4 items). The score ranges from 13 to 91, where a high score indicates a strong SOC. Antonovsky [7,8] did not define boundaries for a normal SOC score but only talked about high and low SOC. A systematic review of the structure of Antonovsky's SOC-13 scale in 127 studies [25] and a population-based study [26] showed that SOC-13 has generally acceptable reliability and validity.

The missing data in our study were substituted separately for each individual who answered at least half of the questions for each component. Only 7 of 227 individuals (3.1%) had one or more items unanswered. At the individual level, the percentage of missing values ranged from 0% (6 items) to 2.2% (item no. 11). Missing substitution for missing value was 3.1% of the SOC total scale and 2.2% for comprehensibility, 0.9% for manageability and 1.3% for meaningfulness.

#### Health-related quality of life

We measured HRQOL using the SF-36. The standard Norwegian version 1 (SF-36) [27] was used. The SF-36 is a generic measure because it assesses health concepts that represent basic human values considered relevant to everyone's functional status and well-being. It is not specific to age, disease or treatment and is widely used in health surveys aiming at measuring physical functioning and social and mental aspects of HRQOL [28,29]. It is also the most commonly used HRQOL instrument [30]. The SF-36 comprises 36 questions (items) along eight dimensions of health: physical functioning (10 items), general health (5 items), mental health (5 items) bodily pain (2 items), role limitation related to physical problems (4 items), role limitation related to emotional problems (3 items), social functioning (2 items) and vitality (4 items). An additional item, reported health transition, notes changes in general health over the past year. The response scores for each dimension are added, and the total is converted to a score between 0 and 100 (highest) [29,31]. A higher score indicates higher HRQOL. The SF-36 has been validated in the general population in Norway [32] and has been used in numerous studies with older people in various settings [33-36], such as measuring the HRQOL among residents in NHs [37,38]. The instrument has demonstrated high reliability (Cronbach's alpha: 0.72–0.94) [38,39] and good construct validity [39] and convergent validity [34]. Using the SF-36 in measuring HRQOL among older NH residents gives the opportunity to compare the results with the general older population and with other relevant studies abroad.

In our study, missing substitution was performed to calculate the score for dimensions when more than 50% of the questions were answered [31]. This was performed for physical functioning (3.1%), role-physical (2.6%) and role-emotional (1.8%). At the individual level, the percentage of missing values for the items in the SF-36 question ranged from 0% (12 items) to 2.6% (item 3).

In the same study, we explored the relationships between HRQOL data and social support [40] and sociodemographic characteristics: living conditions (living in a single room or with another resident), telephone contact with family and friends, hobbies and interests, primary care nurse, duration of stay in the NH and comorbid illnesses [41] this is published elsewhere.

In addition, we investigated length of stay in NH using the same statistical model as in this article.

### Statistical analysis

We performed statistical analysis using SPSS for Windows (Version 14.0, 2005; SPSS) statistical software package. We calculated descriptive statistics for the demographic variables, comorbidity SF-36 subdimensions and the SOC scale.

We checked the reliability of each of the SF-36 subdimensions and SOC by calculating Cronbach's alpha [42].

We used Pearson's correlation coefficient to quantify the level of linear relationship between SOC and the SF-36 dimensions. To adjust for the demographic and comorbidity, we calculated the partial correlation coefficient (partial eta) [43] in a general linear model. This partial correlation coefficient estimates the association between SOC and SF-36 after allowing for the associations with the demographic variables and comorbidity.

We analyzed possible relationships between the SOC variable and the SF-36 subdimensions when controlling for age, sex, marital status, education and comorbidity by using multiple regression in the general linear model procedure of SPSS for Windows (version 14.0). We coded sex, age group, marital status and education as categorical variables and used SOC and comorbidity as continuous covariates. Analysis of residuals showed that one could assume approximate normality for test statistics. The results are stated in term of adjusted regression coefficients for the effect of SOC on each SF-36 subscale. Since the 8 subscales are more or less correlated (max *R *0.544, min *R *0.239), we did not attempt to adjust for inflated Type 1 error. Bonferroni adjustment would give a nominal significance of 0.05/8 = 0.0062 which, however, is thought to be too conservative in this case [43].

We also investigated interactions between the SOC and demographic variables using the general linear model procedure. We generally used the significance level of 0.05.

## Results

### Participants

Table 1 presents the demographic characteristics and comorbidity (FCI) of the 227 respondents. The mean age was 85.4 years (range: 65–102) and the average stay at time of the interview 24 months (range: 6–119). The FCI was 1.9 (median 2.0, standard deviation 1.2, range: 0–6). The most common diagnoses were stroke (including transient ischemic attack): 67 (30%), depression: 40 (18%), congestive heart failure (or heart disease): 38 (17%), and diabetes types 1 and 2: 38 (17%). Generally, men were younger and had higher education, and a higher proportion of men were married.

**Table 1:** Personal characteristics of the 227 respondents

	**Women**		**Men**		**Total**	
	***n***	**%**	***n***	**%**	***n***	**%**
**Sex**	164	72.2	63	27.8	227	100.0
**Age (years)**						
65–74	12	7.3	8	12.7	20	8.8
75–84	48	29.3	30	47.6	78	34.4
85–94	80	48.8	24	38.1	104	45.8
≥ 95	24	14.6	1	1.6	25	11.0
**Marital status**						
Married or cohabiting	13	7.9	25	39.7	38	16.7
Unmarried	27	16.5	8	12.7	35	15.4
Divorced	6	3.7	4	6.3	10	4.4
Widowed	118	72.0	26	41.3	144	63.4
**Education**						
Lowest: primary school	76	46.3	21	33.3	97	42.7
Middle: <3 years after primary school	70	42.7	32	50.8	102	44.9
Highest: ≥ 3 years after primary school	18	11.0	10	15.9	28	12.3
**Comorbidity**						
Yes (FCI^† ^≥ 1)	146	89.0	52	82.5	198	87.2
No (FCI^† ^= 0)	18	11.0	11	17.5	29	12.8

^† ^FCI: Functional Comorbidity Index (Groll et al. 2005) includes 18 diagnoses scored yes = 1 and no = 0 with a maximum score of 18.

Detailed results on the SF-36 scales have been reported elsewhere [41]. On average, residents scored highest on bodily pain (that is, less pain) (mean 71.1, SD 32.7), social functioning (mean 72.9, SD 28.6) and role-emotional functioning (mean 71.7, SD 39.1) and lowest on physical functioning (mean 17.2, SD 20.5). Cronbach's alpha for the SF-36 subscales ranged from 0.91 to 0.72, with physical functioning showing the highest values and social functioning the lowest. The mean SOC of the total study population was 69.1 (SD 12.7), the minimum score being 25 and maximum 90. Men reported higher SOC than women (mean 69.9, standard deviation 11.8), For SOC, Cronbach's alpha was 0.86.

### The relationships between SOC and the SF-36 subdimensions

The sum scores of SOC and all SF-36 subscales were positively correlated (see additional file 1). The strongest correlation was between SOC and mental health score (*r *= 0.61) and the weakest one between SOC and bodily pain (*r *= 0.28). The correlation between SOC and SF-36 subscales did not change substantially after allowing for the association with demographic and comorbidity variables (see additional file 1).

After we adjusted for age group, sex, marital status, educational level and comorbidity, the SOC was still significantly correlated with all SF-36 subscales (see additional file 1). Men and women differed significantly in bodily pain (*P *= 0.006) and physical role limitation (*P *= 0.04). Men scored significantly higher (less pain and less physical role limitation) than women. People with higher education scored higher on bodily pain (less pain, *P *= 0.007), and people with lower education scored higher on social functioning (better social functioning, *P *= 0.005). Multicollinearity was investigated but not found to be a major problem in these data.

We have analyzed length of time as a covariate in the regression model according to each SF-36 subscale. When adjusted for the other covariates (age, sex, martial status, educational level), the variable length of stay was not statistically significant for any subscale. Adjusted *R*^2 ^was unchanged for mental health and vitality and slightly higher for physical functioning, bodily pain and social functioning (0.13 versus 0.12; 0.16 versus 0.15; and 0.19 versus 0.15, respectively). For the other subscales, role-physical, general health and role-emotional, adjusted *R*^2 ^was slightly lower (0.2 versus 0.3; 0.20 versus 0.21; and 0.16 versus 0.15, respectively). Thus, we did not include length of stay in the final model.

### The interaction effects of background variables

For demographic variables and comorbidity that were significantly correlated with any SF-36 scale, we tested for interaction with SOC using the corresponding interaction term in the general linear model. We performed an exploratory examination of the interactions because we suggest, according to the literature [25,44], that the effect of SOC on HRQOL may differ by age, sex and education. No interaction was significant.

## Discussion

The SOC was strongly correlated with SF-36 subdimensions among NH residents after adjusting for education, age, sex, marital status and comorbidity.

In general, the mean SOC-13 score in this study was 69.1. Cole [45] reported a mean score of 65.5 among NH residents aged 72–88 years. Other studies using the SOC-13 scale [10,11,14,44] have reported mean scores between 69.4 and 77.3. These studies were performed on people staying in an acute ward [10] and among people living at home. The mean age varied from 81 (years) to 85 years (and older). Only the study in Norway [44] that included older people (mean age 85 years) receiving home nursing care had results similar to ours.

Our results indicate that SOC is strongly statistically related to SF-36 subdimensions. Our findings could suggest that residents who are able to mobilize the available resources to deal with challenges in everyday life and who experience meaning in doing this may have better HRQOL. Other studies among older people have found similar associations between SOC and the SF-36 mental summary scale [11] and between SOC subscales and the SF-36 physical and mental summary scales [15]. These studies reported no results from each of the 8 subdimensions. Another study [10] showed a bivariate association between SOC and SF-36 subdimensions except for bodily pain and social functioning. In contrast to the study by Ekman et al. [10], our results showed an association between SOC and all the SF-36 subdimensions.

The question remains, however, whether a change in SOC would lead to a corresponding change in HRQOL: that is, would increased SOC ultimately lead to improved HRQOL? The SOC-13 scores varied widely in the study population, with individual scores as low as 25. Some of these very low scores might be related to loss of spouse, relocation and comorbidity. According to the theory, such major life events could lead to temporarily reduced SOC, and these individuals would therefore have the potential to improve their scores [8]. If the strong correlations found indicate that changes in SOC are followed by changes in HRQOL, strengthening the SOC could be important. In this situation, our findings may indicate the importance of investigating measures to strengthen the NH residents' SOC such that the residents' perceived HRQOL could be improved. Although Antonovsky [8] emphasizes that SOC stabilizes in young adulthood, recent empirical findings have shown that SOC changes after intervention [46] and after major life events [47]. However, Antonovsky's opinion was based on theory.

Further, the mental health dimension showed the strongest correlation with SOC. The mental health scale comprises five items ranging from lowest mental health score, associated with marked feelings of nervousness and depressions, to high mental health, associated with peaceful, happy and calm feelings [31]. A systematic review of the SOC-13 and its relationship to health [48] found that SOC is strongly related to mental health. Another study has discussed whether SOC and mental health are aspects of the same global construct [49]. However, based on confirmatory factor analysis and structural equation modeling, Eriksson & Lindstrom [48] emphasize that SOC and mental health are two independent but correlated constructs. An essential finding in our study is the strong statistically relationship between SOC and SF-36 mental health dimension for NH residents. Because the design was cross-sectional, we cannot conclude on the direction, and a bidirectional effect is possible.

Moreover, our results showed weaker correlations between the physical functioning subdimension and SOC. The physical functioning subdimension comprises 10 items ranging from lowest physical health score associated with marked limit in performing all physical activities including bathing and dressing due to health, to high physical health, associated with performing all types of physical activities without limitations to health [31]. Eriksson & Lindstrom's [48] review of SOC and health also found this overall. As Antonovsky [8] describes SOC, suggesting that an individual's SOC is more directly correlated with psychosocial reactions than physical behavior, our finding is reasonable. Antonovsky [8] stated that, if the demands become less comprehensible or manageable, then the person accidentally or permanently restricts the boundary for what is most important in his or her life. It could mean that people living in an NH set other boundaries in life that are more important and different. For example, these NH residents reported a low score on the physical functioning subdimension, indicating limited performance and physical activities. Residents who no longer have physical ability but have mental ability can find other areas in life that are meaningful: the disability paradox. Albrecht & Devlieger [50] confirmed the existence of the disability paradox in a study among respondents who had moderate to serious disability. Despite disability, these respondents reported excellent or good quality of life.

Beyond that, the NH residents in our study, despite reduced capacity and adversity, have adapted their living conditions and coped with diseases or impairments. Further, physical impairment can be understood as salutogenesis (in terms of positive adaptation and resolution to stress) rather than pathogenesis [8,50]. Thus, the stronger the SOC, the more flexibility concerning the areas that are the most important [8,10].

Possible improvements in clinical practice in NHs could be guided by the use of the three SOC components comprehensibility, manageability and meaningfulness to strengthen residents' SOC. In relation to comprehensibility, it is important that residents are informed about and understand the nature of their care. For example, health care professionals can make living conditions in NHs more comprehensible and predictable for the residents by providing health care information and health care in a consistent way. Manageability could be enhanced by having family and health care professionals provide resources such as social support [51]. Health care professionals can make families aware of the residents' resources and help the residents to use these resources. In addition, families may also be a good source of information concerning the residents' resources such as previous interests, hobbies etc. Further, health care professionals need to be aware of how care plans may contribute to the residents' need for and desire to feel a sense of control over their daily lives. When residents are in control of their lives, they feel more satisfied with life [52]. Having a sense of control over situations such as going to bed, eating and care routines may contribute to the experience of manageability. Meaningfulness means having the motivation and desire to cope with internal and external stimuli [8] and, for Antonovsky [8], meaningfulness is the most important aspect in strengthening SOC. Antonovsky [8] suggested four areas in which people need to invest if they want to maintain a sense of meaningfulness: feelings, interpersonal relationships, employment and existential value. Health care professionals could facilitate meaningfulness for the residents by supporting them in maintaining their close relationships and by providing emotional support, and providing opportunities for activities such as occupational therapy and participation in the political, cultural and religious arenas. In this way, health care professionals can encourage the residents to engage in activities in the NH and in activities they previously valued but had to give up after being admitted to the NH. This may contribute to a sense ofmeaningfulness for the residents that, in turn, strengthen their SOC.

### Methodological issues

Several limitations of this study should be considered when interpreting the results. The sample is based on relatively strict inclusion criteria. Of the 2042 NH residents, 252 fulfilled the inclusion criteria. Beyond that, the participation rate was high (90%). Dementia was not diagnosed as part of this study. To reach the target population, we took a rather pragmatic position when including NH residents with CDR scores of 0 and 0.5. In our setting, CDR of 0.5 is understood as senescent forgetfulness: these participants have minor memory problems that do not impair daily functioning and are capable of normal conversation. The result is therefore applicable to subjects living in NHs in Bergen who fulfill the inclusion criteria.

Few data were missing on the SF-36. The missing data were related to questions concerning physical functioning (strenuous activity) and role-physical (problems with work and daily activities). As reported in other studies [33,34], these questions are generally not relevant for NH residents. Nevertheless, other studies have suggested that, in an interview setting, the SF-36 is suitable for use among older people, whether living at home [39] or in an NH [38]. Very few data were missing from the SOC-13, and generally the respondents did not find the questionnaires difficult to answer.

Other measures that might help to understand SOC include social support, because this is a resource for shaping the SOC [7,8]. We have previously analyzed data from the same study with social support and SOC related to HRQOL [40]. The results showed that SOC significantly contributed to the explained variance in HRQOL independent of social support. The effect of social support on HRQOL disappeared when SOC was controlled for only one of the three social support subdimensions.

Further, data about stress factors within NHs and specific evaluation of the reasons for recovery in NH could also be important to investigate in relation to SOC and HRQOL.

Finally, due to the cross-sectional nature of the study, we can only interpret the results as associations, although the regression model applied implicitly defines SOC as explaining HRQOL. A bidirectional effect is possible: an increase in SOC might result in better HRQOL or residents who have better HRQOL might also have strong SOC. Nevertheless, Antonovsky [7] suggested that SOC predicts well-being, and studies have shown that SOC and HRQOL are significantly related [48].

## Conclusion

Our study found small changes when we adjusted the relationship between the SOC-13 and SF-36 subdimensions for demographic variables, age group, marital status, education and comorbidity. This indicates that the relationship varies little between subgroups, that the SOC-13 is strongly statistically associated with the SF-36 subdimensions and that the SOC-13 may be useful for this kind of study. Moreover, our findings give credence to Antonovsky's hypothesis on the relationship between SOC and well-being.

Although there is some literature on the relationship between SOC and HRQOL among older people in general, our findings have shown that the SOC-13 is strongly related to SF-36 subdimensions among older people living in NHs. To our knowledge, this is the first attempt to demonstrate this relationship among mentally intact NH residents. Health care professionals need to recognize that SOC is associated with HRQOL and that strengthening residents' SOC will improve their HRQOL. Professionals can contribute to strengthening the residents' SOC by identifying their previous strengths and the internal and external resources they currently have available and helping them to use these despite any limitations the residents may have. Further, concerning care plans, professionals could provide health care information to residents in a way that is easy for them to understand. Professionals could also encourage residents to engage in the kind of everyday activities that are meaningful for them. Health care professionals play a key role in helping the older residents to maximize these opportunities which, in turn, may improve their HRQOL. Further, an intervention study is needed to determine whether SOC contributes to higher HRQOL.

## Abbreviations

SOC: Sense of coherence; HRQOL: Health-related quality of life; NH: Nursing home; SOC-13: Sense of Coherence Scale; SF-36: SF-36 Health Survey; NHs: Nursing homes; CDR: Clinical Dementia Rating; FCI: Functional Comorbidity Index.

## Competing interests

The authors declare that they have no competing interests.

## Authors' contributions

JD designed the study, carried out the survey, collected the data and drafted the manuscript. HAN participated in the design of the study and revised it critically for important intellectual content. GEE, in close cooperation with JD, planned and performed the data analysis. MB and MWN revised the manuscript critically for important intellectual content. GKN participated in the design of the study and revised the manuscript critically for important intellectual content. All authors commented on drafts of the manuscript and read and approved the final manuscript.

## Supplementary Material

Additional file 1 Analysis of covariance of each subscale of SF-36 (*n *= 227) with respect to SOC adjusted for sex, age group, marital status, educational level and comorbidity. Click here for file
