# Supplementary material for: Sense of coherence as a resource in relation to health-related quality of life among mentally intact nursing home residents – a questionnaire study
Source: Health Qual Life Outcomes. 2008 Oct 21;6:85. doi: 10.1186/1477-7525-6-85 (PMC2607268; doi:10.1186/1477-7525-6-85)
Supplement: Additional file 1 — Analysis of covariance of each subscale of SF-36 (n = 227) with respect to SOC adjusted for sex, age group, marital status, educational level and comorbidity. [file 1477-7525-6-85-S1.doc]

**Table 2: Analysis of covariance of each subscale of SF-36 (*n* = 227) with respect to SOC adjusted for sex, age group, marital status, educational level and comorbidity**

SOC = sense of coherence, b = regression coefficient, CI = confidence interval.

| **SF-36 subscales** | **PF** | **RP** | **BP** | **GH** | **V** | **SF** | **RE** | **MH** |
| --- | --- | --- | --- | --- | --- | --- | --- | --- |
| **Covariates** | b 95 % CI | b 95 % CI | b 95 % CI | b 95 % CI | b 95 % CI | b 95 % CI | b 95 % CI | b 95 % CI |
| **Intercept** | –14.5 (–33.7, 4.6) | –30.9 (–70.6, 8.8) | 28.2 ( –1.8, 58.1) | 5.4 (–13.3, 24.0) | –15.2 (–34.3, 3.8) | 12.2 (–14.1, 38.4) | –7.0 ( 42.8, 28.8) | 2.1 ( 14.3, 18.4) |
| **Sex** |  |  |  |  |  |  |  |  |
| Male | 4.8 ( –1.4, 11.1) | 13.9 ( 1.0, 26,9) | 13.9 ( 4.1, 23.7) | 0.7 ( –5.5, 6.8) | 1.3 ( –4.9, 7.5) | –2.3 (–10.8, 6.3) | –5.0 (–16.8, 6.7) | –0.2 ( –5.5, 5.2) |
| Female | reference | reference | reference | reference | reference | reference | reference | reference |
| ***P*** |  | **0.036** | **0.006** |  |  |  |  |  |
| **Age (years)** |  |  |  |  |  |  |  |  |
| 65–74 | –8.1 (–20.2, 4.1) | 16.4 ( –8.6, 41.5) | 14.8 ( –4.1, 33.6) | –5.5 (–17.3, 6.4) | 13.7 ( 1.7, 25.7) | 4.4 (–12.2, 20.9) | 3.2 (–19.7, 6.0) | 3.5 ( –6.7, 13.8) |
| 75–84 | 3.9 ( –5.3, 13.2) | 2.8 (–16.4, 22.0) | 1.9 (–12.6, 16.3) | –0.1 ( – 9.2, 8.9) | 4.9 ( –4.2, 14.1) | 7.1 ( –5.6, 19.8) | 2.5 (–14.8, 9.8) | –2.5 (–10.4, 5.3) |
| 85–94 | –3.8 (–12.5, 5.0) | 4.7 (–13.4, 22.8) | 7.1 ( –6.6, 20.7) | –2.4 (–10.9, 6.2) | 2.3 ( –6.4, 10.9) | 8.9 ( –3.0, 20.9) | 4.4 (–11.9, 0.7) | 0.7 ( –6.7, 8.2) |
| ≥ 95 | reference | reference | reference | reference | reference | reference | reference | reference |
| ***P*** | **0.023** |  |  |  |  |  |  |  |
| **Marital status** |  |  |  |  |  |  |  |  |
| Married | –3.7 (–11.5, 4.1) | –7.6 (–23.7, 8.5) | –6.2 (–18.4, 5.9) | –4.9 (–12.5, 2.7) | –5.7 (–13.4, 2.0) | –3.6 (–14.3, 7.0) | 3.7 (–10.9, 18.3) | 1.5 ( 5.1, 8.1) |
| Unmarried | –1.2 ( –8.7, 6.2) | 12.7 ( –2.7, 28.2) | 11.6 ( 0.0, 23.3) | 2.6 ( –4.7, 9.9) | –1.5 ( –5.9, 8.9) | 7.1 ( –3.0, 17.3) | 16.9 ( 3.0, 30.9) | 1.1 ( –5.3, 7.4) |
| Divorced | –9.9 (–23.1, 3.4) | 10.5 (–16.9, 37.9) | –8.6 (–29.3, 12.1) | – 4.1 (–17.1, 8.8) | 2.1 (–11.0, 15.3) | –12.3 (–30.0, 5.8) | 6.6 (–18.2, 31.4) | 0.1 (–11.2, 11.3) |
| Widowed | reference | reference | reference | reference | reference | reference | reference | reference |
| ***P*** |  |  |  |  |  |  |  |  |
| **Educational**  **level** |  |  |  |  |  |  |  |  |
| Lowest | 1.0 ( 7.3, 9.3) | –0.9 (–18.0, 16.2) | –11.6 (–24.5, 1.3) | 3.3 ( –4.8, 11.4) | 1.3 ( –6.9, 9.5) | 0.4 ( 11.0, 11.7) | –9.9 (–25.3, 5.6) | –1.2 ( –8.3, 5.8) |
| Middle | – 0.5 ( –8.6, 7.8) | –13.0 (–29.9, 4.0) | –19.7 (–32.5, –6.9) | –2.7 (–10.7, 5.4) | 0.3 ( –7.9, 8.4) | –11.6 (–22.8, –0.4) | –11.4 (–26.7, 3.8) | –3.4 (–10.4, 3.6) |
| Highest | reference | reference | reference | reference | reference | reference | reference | reference |
| ***P*** |  |  | **0.007** |  |  | **0.005** |  |  |
| **Comorbidity** | – 1.4 ( –3.5, 0.8) | 1.5 ( –2.9, 5.9) | –2.5 ( –5.8, 0.8) | –0.6 ( –2.7, 1.5) | 0.9 ( –1.2, 3.0) | 1.0 ( –1.9, 3.8) | –1.4 ( –5.3, 2.6) | 0.1 ( –1.7, 1.9) |
| ***P*** |  |  |  |  |  |  |  |  |
| **SOC** | 0.5 ( 0.3, 0.7) | 1.2 ( 0.8, 1.6) | 0.8 ( 0.4, 1.1) | 0.8 ( 0.6, 1.0) | 0.7 ( 0.5, 0.9) | 0.8 ( 0.6, 1.1) | 1.2 ( 0.9, 1.6) | 1.0 ( 0.8, 1.2) |
| ***P*** | **<0.0001** | **<0.0001** | **<0.0001** | **<0.0001** | **<0.0001** | **<0.0001** | **<0.0001** | **<0.0001** |
| **Adjusted *R*^2^** | 0.12 | 0.13 | 0.15 | 0.21 | 0.18 | 0.15 | 0.15 | 0.36 |
| **Partial eta** | 0.32 | 0.36 | 0.32 | 0.46 | 0.44 | 0.37 | 0.40 | 0.61 |
| **Pearson *r**** | 0.32 | 0.33 | 0.28 | 0.55 | 0.42 | 0.35 | 0.40 | 0.62 |

Pearson correlation and partial correlations were significant at 0.01 (two-tailed) for all SF-36 subdimensions: PF, physical functioning; RP, role–physical; BP, bodily pain; GH, general health;

V, vitality; SF, social functioning; RE, role–emotional; MH, mental health.
